# Proteomics of fibrin amyloid microclots in long COVID/post-acute sequelae of COVID-19 (PASC) shows many entrapped pro-inflammatory molecules that may also contribute to a failed fibrinolytic system

**DOI:** 10.1186/s12933-022-01623-4

**Published:** 2022-09-21

**Authors:** Arneaux Kruger, Mare Vlok, Simone Turner, Chantelle Venter, Gert Jacobus Laubscher, Douglas B. Kell, Etheresia Pretorius

**Affiliations:** 1 Department of Physiological Sciences, Faculty of Science, Stellenbosch University, Private Bag X1 Matieland, Stellenbosch, 7602 South Africa; 2 Central Analytical Facility, Mass Spectrometry Stellenbosch University, Tygerberg Campus, Room 6054, Clinical Building, Francie Van Zijl Drive, Tygerberg, Cape Town, 7505 South Africa; 3 Mediclinic Stellenbosch, Stellenbosch, 7600 South Africa; 4 Department of Biochemistry and Systems Biology, Institute of Systems, Molecular and Integrative Biology, Faculty of Health and Life Sciences, University of Liverpool, Liverpool, L69 7ZB UK; 5 The Novo Nordisk Foundation Centre for Biosustainability, Technical University of Denmark, Kemitorvet 200, 2800 Kongens Lyngby, Denmark

**Keywords:** Long COVID, Microclots, Platelet hyperactivation, von Willebrand factor, Kallikrein, Platelet factor 4, Antibodies, Failed fibrinolysis

## Abstract

**Background:**

Post-acute sequelae of COVID-19 (PASC), also now known as long COVID, has become a major global health and economic burden. Previously, we provided evidence that there is a significant insoluble fibrin amyloid microclot load in the circulation of individuals with long COVID, and that these microclots entrap a substantial number of inflammatory molecules, including those that might prevent clot breakdown. Scientifically, the most challenging aspect of this debilitating condition is that traditional pathology tests such as a serum CRP (C-reactive protein) may not show any significant abnormal inflammatory markers, albeit these tests measure only the soluble inflammatory molecules. Elevated, or abnormal soluble biomarkers such as IL-6, D-Dimer or fibrinogen indicate an increased risk for thrombosis or a host immune response in COVID-19. The absence of biomarkers in standard pathology tests, result in a significant amount of confusion for patients and clinicians, as patients are extremely sick or even bed-ridden but with no regular identifiable reason for their disease. Biomarkers that are currently available cannot detect the molecules present in the microclots we identified and are therefore unable to confirm their presence or the mechanisms that drive their formation.

**Methods:**

Here we analysed the protein content of double-digested microclots of 99 long COVID patients and 29 healthy controls. The patients suffering from long COVID reported their symptoms through a questionnaire completed by themselves or their attending physician.

**Results:**

Our long COVID cohort’s symptoms were found to be in line with global findings, where the most prevalent symptoms were constant fatigue (74%,) cognitive impairment (71%) and depression and anxiety (30%). Our most noteworthy findings were a reduced level of plasma Kallikrein compared to our controls, an increased level of platelet factor 4 (PF4) von Willebrand factor (VWF), and a marginally increased level of α-2 antiplasmin (α-2-AP). We also found a significant presence of antibodies entrapped inside these microclots.

**Conclusion:**

Our results confirm the presence of pro-inflammatory molecules that may also contribute to a failed fibrinolysis phenomenon, which could possibly explain why individuals with long COVID suffer from chronic fatigue, dyspnoea, or cognitive impairment. In addition, significant platelet hyperactivation was noted. Hyperactivation will result in the granular content of platelets being shed into the circulation, including PF4. Overall, our results provide further evidence of both a failed fibrinolytic system in long COVID/PASC and the entrapment of many proteins whose presence might otherwise go unrecorded. These findings might have significant implications for individuals with pre-existing comorbidities, including cardiovascular disease and type 2 diabetes.

## Introduction

Post-acute sequelae of COVID-19 (PASC), now commonly referred to as long COVID, has quickly become a major global health and economic burden [1–4]. SARS-CoV-2 typically makes use of the cell surface receptor angiotensin-converting enzyme 2 (ACE2), to enter target cells. ACE2 receptors are expressed on the surface of lung epithelial cells, enterocytes of the small intestine and are also present in arterial and venous endothelial cells as well as arterial smooth muscle cells of multiple organs [5]. Viral replication leads to cellular damage and the release of pro-inflammatory alarmins due to cell injury and death [6]. The complement system may be activated by locally formed immune complexes which would inevitably boost the inflammatory response [7]. The activation of the complement cascade directly leads to endothelial damage and C3a and C5a formation recruits leucocytes that are responsible for a substantial local release of pro-inflammatory cytokines such as interleukin IL-1, IL-6, IL-8 and interferon-γ [8]. Fabio Ciceri and co-authors suggested the use of the term MicroCLOTS (microvascular COVID-19 lung vessels obstructive thromboinflammatory syndrome) [9]. They used this term as an atypical acute respiratory distress syndrome because of lung damage, primarily explained by dramatic alveolar endothelial damage leading to a progressive endothelial pulmonary syndrome with microvascular thrombosis [9]. In the acute phase of COVID-19, patients present with multi-organ symptoms [10] and this phase is characterized by acute clinical pathologies, including various coagulopathies that may be accompanied by hypercoagulation and platelet hyperactivation [11–17]. If these coagulopathies are not adequately treated during the acute phase of the disease, possible tissue hypoxia and impaired oxygen exchange may linger for months. The resulting underlying pathologies can directly perpetuate lingering long COVID symptoms that include fatigue or muscle weakness, dyspnoea, cognitive impairment, insomnia, and anxiety or depression.

We recently provided evidence, based on a relatively small cohort of patients, that individuals with long COVID present with a significant fibrin amyloid microclot load in their circulation and that these persistent microclots are resistant to fibrinolysis [1]. Microclots as described further in this paper, will refer to microscopic (fibrin-amyloid-containing) clots present in the plasma of recruited individuals and not MicroCLOTS, the syndrome as suggested by Ciceri and co-authors [9]. In our previously mentioned work, we used techniques including proteomics and fluorescence microscopy to study plasma samples from healthy individuals, individuals with type 2 diabetes mellitus (T2DM), those with acute COVID-19, and those with long COVID symptoms. Plasma samples from long COVID patients still contained large amounts of anomalous (amyloid) microclots that are resistant to fibrinolysis (compared to the plasma from controls and T2DM), even after trypsinisation. We could solubilize the microclots only after a second trypsinization step. The most noteworthy inflammatory molecules that were trapped in the solubilized microclots present in plasma of long COVID patients, versus the equivalent volume of fully digested plasma fluid of control samples, were α(2)-antiplasmin (α2AP), various fibrinogen chains, von Willebrand factor (VWF), and Serum Amyloid A (SAA). In addition to the natural resistance of protein amyloid structures to proteolysis [18] these trapped molecules could indeed result in fibrinolytic-resistant pellet microclots [1, 19].

We have now embarked on a considerably more extensive analysis of these microclot contents. We used proteomics to study blood samples from 99 long COVID patients and 29 healthy controls. There is increasing evidence that autoantibodies may be involved in the lingering symptoms of these patients [20–22]. Thus, in addition to molecules of interest that are related to clotting, we also focused on the presence of antibodies or autoantibodies that might be associated with or trapped inside the fibrinolysis-resistant microclots.

## Materials and methods

### Ethical clearance

Ethical clearance for the study was obtained from the Health Research Ethics Committee (HREC) of Stellenbosch University (South Africa) (references: B21/03/001_COVID-19, project ID: 21,911 (long COVID registry) and N19/03/043, project ID 9521 with yearly re-approval). Participants were either recruited via the long COVID registry or identified from our clinical collaborators’ practice. The experimental objectives, risks, and details were explained to volunteers and informed consent were obtained prior to blood collection. Strict compliance to ethical guidelines and principles Declaration of Helsinki, South African Guidelines for Good Clinical Practice, and Medical Research Council Ethical Guidelines for Research were kept for the duration of the study and for all research protocols.

### Sample demographics and considerations

Blood was collected from 29 volunteers (9 males; 20 females; median age 52 [range 41–57]) to serve as controls. Control patients did not smoke or suffer from coagulopathies, were not pregnant and only 1 volunteer suffered from cardiovascular disease. Those suffering from hypertension or hyperlipidaemia, were well controlled on treatment and none of our controls took anticoagulation medication. Initially, we included 99 long COVID patients in the study (30 males, 69 females.) Liquid chromatography and mass spectrometry were performed on all of the samples and data collected. 66 of the long COVID samples were found to be “high responders” and 24 “low responders” (please refer to the mass spectrometry data analysis section for an in-depth explanation of the classification.) Mass spectrometry data analysis was only carried out on the high responder long COVID samples. The median age of high responders was 51 [40–60] (21 males; 45 females) and the median age of low responders 45 [31–58] (5 males; 19 females.) Healthy participants, patients or their attending physicians completed a questionnaire, either as part of the long COVID registry or when they visited their attending clinician. The questionnaire contained information regarding their age, gender, pre-existing comorbid conditions, chronic medication, information regarding the onset, diagnosis, and severity of their acute COVID-19 illness, self-reported long COVID symptoms, as well as their COVID-19 vaccination history (where available).

### Blood sample collection

Either a qualified phlebotomist or medical practitioner drew blood samples. Blood samples were collected in 4.5 mL sodium citrate (3.2%), 4 ml lithium heparin (75 USP units) and 5 ml rapid serum (thrombin-based clot activator) tubes (BD Vacutainer®, 369714)), via venepuncture, adhering to the standard sterile protocol. On the day of blood collection, natriuretic peptide tests measuring levels of BNP or NT-proBNP, serum ferritin and Interleukin-6 (IL-6) levels were analysed by a pathology lab. NT-proBNP were analysed to determine that our cohort did not have underlying heart failure that might falsely suggest coagulopathies are due to long COVID. If NT-proBNP levels were not available, the attending clinician ensured that the patients had normal echocardiograms. Whole blood (WB) was centrifuged at 3000x*g* for 15 min at room temperature and the supernatant platelet poor plasma (PPP) samples were collected in 1.5 mL Eppendorf tubes and stored at – 80 °C.

### Double trypsin digestion protocol to solubilize microclots in PPP

Stored PPP was used to determine protein concentration using nanodrop technology where a 20 × dilution was made with 10 mM ammonium bicarbonate and protein concentration determined using a nanodrop spectrophotometer (Thermo Fischer) and measuring the absorbance at 280 nm. The samples were then standardised to the lowest protein concentration and 50 µg total protein removed for digestion (25 µL). For the first trypsin digestion step, 50 µg of protein per sample was placed in a tube and 25 µL ammonium bicarbonate and 1 µg of trypsin was added to a final volume of 50 µL. The samples were incubated over night at 37 °C.

For the second trypsin digestion step, the samples were centrifuged the next day at 16,000*g* for 10 min (volume in each sample were still 50 µL). Next, 42 µL of supernatant were removed carefully to not do not disturb the microclots at bottom of tube. To the remaining 8 µL, 10 µL of 250 mM Triethyamonium bicarbonate (TEAB) (ThermoFisher) containing 5 mM tris carboxyethyl phosphine (TCEP) (Sigma), was added. The samples were vortexed for 5 s followed by incubation of 10 min at 90 °C. Samples where then incubated for a further 50 min at 60 °C, followed by a cooling-down step. Cysteine residues were blocked using 10 mM (final concentration) methyl methanethiosulfonate (MMTS) (we added 1 µl into each sample). Samples were left at room temp for 30 min. Thereafter, 20 µL of 50% (Sigma) methanol was added and samples were vortexed for 5 s (to denature last intact proteins that might remain). Now 10 µL of trypsin solution in 100 mM TEAB (containing 1 µg of trypsin) (Thermo) was added to each sample followed by incubation for 18 h at 37 °C. Samples were then acidified with 5 µL trifluoroacetic acid (TFA) (Sigma) of which the final concentration of 0.5% was used to precipitate trypsin. Samples where centrifuged at 16,000*g* to pellet the trypsin precipitate and the supernatant removed and placed into HPLC inserts. Samples were allowed to evaporate to dryness under vacuum (roto-evaporator) followed by resuspending in 20µL loading buffer consisting of 2% acetonitrile (Burdick and Jackson) in deionized water containing 0.1% Formic acid (Sigma) spiked with Biognosys 11 indexed retention times standards (Biognosys.) The dissolved samples were loaded directly in the autosampler set to 4C.

### Liquid chromatography of digested microclots

Liquid chromatography was performed on a Thermo Scientific Ultimate 3000 RSLC [23, 24] equipped with a 20 mm × 100 µm C_18_ trap column (Thermo Scientific) and a CSH 25cmx75µm 1.7 µm particle size C_18_ column (Waters) analytical column. The solvent system employed was loading: 2% acetonitrile:water; 0.1% FA; Solvent A: water; 0.1% FA and Solvent B: 100% acetonitrile, 0.15% FA. Methods were previously described in [1]. Separation was effected using a linear gradient from 2 B to 30% B over 65 min followed by 30 B to 45% B from 65 to 80 min. The sample was loaded onto a trap column at 2 µL/min before switching to the analytical column at three minutes with a flow rate of 300 nL/min. Chromatography was performed at 45 °C and the outflow delivered to the mass spectrometer through a stainless-steel nano-bore emitter.

### Mass spectrometry of digested microclots

Mass spectrometry was performed using a Thermo Scientific Fusion mass spectrometer equipped with a Nanospray Flex ionization source. Data was collected in positive ion mode with spray voltage set to 1.8 kV and ion transfer capillary set to 275 °C. The mass spectrometer auto-calibration was activated using polysiloxane ions at m/z = 445.12003. MS^1^ scans were performed using the orbitrap detector set at 120,000 resolution over the scan range 375–1500 with AGC target at 2 E^4^ and maximum injection time of 50 ms. Data were acquired in profile mode. MS^2^ acquisitions were performed using monoisotopic precursor selection for ion with charges + 2 to + 7  with error tolerance set to ± 10 ppm. Precursor ions were excluded from fragmentation once for a period of 60 s. Precursor ions were selected for fragmentation in HCD mode using the quadrupole mass analyser with HCD energy set to 30%. Quadrupole isolation was set at m/z = 1.2. Fragment ions were detected in the Orbitrap mass analyser set to 30,000 resolution. The AGC target was set to 5E4 and the maximum injection time to 100 ms. The data was acquired in centroid mode. Samples were randomised by using a randomization schedule. Technical replicates are not feasible in an experiment with a large sample size such as this.

### Mass spectrometry data analysis

The raw files generated by the mass spectrometer were imported into SearchGUI [25] and processed using the MsGF + algorithm. Database interrogation was performed against the 2019-nCOVpFASTA database [26]. Semi-tryptic cleavage with 2 missed cleavages was allowed for. Precursor mass tolerance was set to 10 ppm and fragment mass tolerance set to 0.02 Da. Deamidation (NQ), oxidation (M) and Phosho-ST were allowed as dynamic modifications and methylthio of C as a fixed modification. Data was imported into Skyline and the extracted ion chromatograms of positively identified peptides summated as indication of relative protein abundance. Spectral data were evaluated in Skyline and the proteomics sample was divided into high and low responders. Samples where 90% of all the protein intensity level read outs (or the area under the peak) were above the 3rd quartile when compared to the protein levels observed in our other samples, were classified as “high responders". If 90% of all the protein intensity level read outs were below the 1st quartile when compared to the proteins in other samples, they were classified as “low responders.” For example, if there were 100 proteins identified but 90 of those proteins had concentrations/responses that were below the 1st quartile compared to the other samples in the sample set, the patient was classified as a low responder. Low responders were excluded from further analysis. We ensured that it was not attributable to instrument performance and batch effects were also excluded. The search results were imported into Scaffold Q + for further validation (www.proteomesoftware.com) and statistical testing. The (FDR) false discovery rate was set to 1%. Normalization was selected in Scaffold Q + with a weighted spectra quantitation method and missing values were processed by the algorithm contained within the software. A Fischer’s exact test (F-test) was performed on the dataset and Benjaminin-Hochberg multiple testing correction applied. This resulted in a p-value of < 0.012. For immunoglobulin specific searches the FASTA files were obtained from uniprot (www.uniprot.org) by searching the database for human immunoglobulins and downloading the returned protein sequences. Protein parsimony for immunoglobulin searches was handled by peptide and Protein Prohpet algorithms within Scaffold. The immunoglobulins listed later in the paper (as shown in Table 3 and Table 4,) were cross referenced on THE INTERNATIONAL IMMUNOGENETICS INFORMATION SYSTEM®’s IMGT/LIGM-DB database (nucleotide sequences of IG and TR from 360 species.) The citable accession numbers of the immunoglobulins were used to determine the EMBL^i^ reference number to search for the particular immunoglobulin on the database: https://www.imgt.org/ligmdb/ Our proteomics data will be deposited in an international repository such as PRIDE (EBI) for public access.

### PPP and the detection of amyloid fibrin(ogen) protein and anomalous micro-clotting

Fluorescence microscopy of microclot formation in PPP was performed on some of our samples as described in our previous papers [17]. PPP were exposed to the fluorescent amyloid dye, Thioflavin T (ThT) (final concentration: 0.005 mM) (Sigma-Aldrich, St. Louis, MO, USA) for 30 min (protected from light) at room temperature [18, 27–30]. After incubation, 3 µL stained PPP was placed on a glass slide and covered with a coverslip. The excitation wavelength band for ThT was set at 450 to 488 nm and the emission at 499 to 529 nm and processed samples were viewed using a Zeiss Axio Observer 7 fluorescent microscope with a Plan-Apochromat 63x/1.4 Oil DIC M27 objective (Carl Zeiss Microscopy, Munich, Germany).

### Platelet pathology / evaluation

Haematocrit samples of a few of our patients in the cohort were exposed to the two fluorescent markers, CD62P (PE-conjugated) (platelet surface P-selectin) (IM1759U, Beckman Coulter, Brea, CA, USA) and PAC-1 (FITC-conjugated) (340507, BD Biosciences, San Jose, CA, USA) (17). CD62P is a marker for P-selectin that is either found on the membrane of platelets or inside them. PAC-1 identifies platelets through marking the glycoprotein IIb/IIIa (gpIIb/IIIa) on the platelet membrane. To study platelet pathology, 4 µL CD62P and 4 µL PAC-1 was added to 20 µL haematocrit, followed by incubation for 30 min and protected from light at room temperature. The excitation wavelength band for PAC-1 was set at 450 to 488 nm and the emission at 499 to 529 nm and for the CD62P marker it was 540 to 570 nm and the emission 577 to 607 nm [17]. Samples were viewed using a Zeiss Axio Observer 7 fluorescent microscope with a Plan-Apochromat 63x/1.4 Oil DIC M27 objective (Carl Zeiss Microscopy, Munich, Germany).

### Statistics

Statistical analysis was done using Graphpad Prism 8 (version 8.4.3). All data were subjected to Shapiro-Wilks normality tests. An unpaired T-test was performed on parametric data with the data expressed as mean ± standard deviation, whereas the Mann–Whitney U test was used on unpaired non-parametric data and the data expressed as median [Q1–Q3] (all two-tailed).

## Results

Individuals suffering from long COVID in South Africa, were invited to fill in an online South African long COVID registry and they could, in addition, opt to be contacted to provide a blood sample (ethics number for registry: B21/03/001_COVID-19, project ID: 21911). Individuals that served as controls, were also recruited for a blood sample and filled in consent forms as per ethics number N19/03/043, project ID 9521.

The demographics, serum ferritin and IL-6 levels performed on the day of blood collection, self-reported symptom analysis and comorbidities before suffering from acute COVID-19 of our long COVID patients and controls are shown in Table 1 below. Our results follow trends reported on previously [34, 35]. Interestingly, previously it has been reported that misfolded protein aggregates behave as infectious agents [36]. Of interest is that 24% of the participants in our current long COVID cohort, reported to have hypertension and 19% hyperlipidaemia, prior to contracting COVID-19. 24% of our control patients suffered from hypertension, 24% from hyperlipidaemia and 3% (1 volunteer) from cardiovascular disease. The most prevalent long COVID symptoms of the current cohort were noted as constant fatigue (74%) and cognitive impairment [reported by the patients as “brain fog”, forgetfulness, or poor concentration (71%.)] We wish to emphasize the fact that our control patients did not suffer from long COVID and were asymptomatic. The severity of acute COVID-19 disease in our patients suffering from long COVID varied from mild or moderate to severe requiring oxygen or ventilation. We analysed our long COVID cohort and divided them into groups of severity as per the WHO clinical progression scale [31]. Patients with mild disease included asymptomatic patients as well as patients with clinical symptoms but were not hospitalized; patients with moderate disease were hospitalized and included patients that didn’t receive oxygen or received oxygen via face mask or nasal prongs and patients with severe disease were hospitalized and received high flow oxygen or were mechanically ventilated. 71% had mild disease, 5% moderate disease, 17% had severe disease and 7% were unknown (patients that we lost to follow up.) 21% of the long COVID cohort were hospitalized, 72% did not require admission and 7% were unknown. The duration of symptoms from diagnosis to the taking of blood samples from the long COVID cohort was 221 ± 99 days.

**Table 1:** Demographics of long COVID patients and controls

Demographics	
Median age of healthy individuals (n = 29)	52 [41–57]
Median age of long COVID (high responders) (n = 66)	51 [40–60]
Median age of long COVID (low responders) (n = 24)	45 [31–58]

Serum Ferritin (µg L^−1^)	
P-value (Mann–Whitney U test; unpaired non-parametric data expressed as median [Q1–Q3] of high responders)	0.80
Median of healthy individuals (n = 29)	120 [40–230]
Median of long COVID (63 of 66) high-responder patients	107 [61.0–179.5]
Median of long COVID of the low responder patients (24 of 24) patients. [p-Value = 0.36]	74 [33.5–153.5]

Interleukin-6 (pg ml^−1^)	
P-value (Mann–Whitney U test; unpaired non-parametric data expressed as median [Q1 – Q3] of high responders)	0.04
Median of healthy individuals (28 of 29 samples)	1.9 [1.1–2.5]
Median of long COVID (63 of 66) high-responder patients	2.3 [1.5–3.5]
Mean of long COVID of the low responder patients (24 of 24 patients) [p-Value = 0.65]	1.9 [± 0.6]

Hospitalization (percentage / %)	
Yes	21
No	72
Unknown	7

*WHO severity (percentage / %)*	
Mild	71
Moderate	5
Severe	17
Unknown	7

Co-morbidities of long COVID patients (N = 99) versus our healthy controls (N = 29)		
Co-morbidity	% in 99 long Covid patients	% in 29 control patients
Hypertension	24	24
Hyperlipidaemia	19	24
Type 2 diabetes mellitus	6	0
Rosacea	4	3
Thrombosis (previous blood clots)	3	0
Cardiovascular disease	3	3
Psoriasis	2	3
Rheumatoid arthritis	2	0
Previous stroke	1	3
Previous myocardial infarction	1	0

Lingering symptoms in long COVID patients (N = 99) Duration of symptoms from diagnosis to taking of blood samples (days:) 221 ± 99	
Lingering symptoms	% in 99 long Covid patients
Constant fatigue	74
Cognitive impairment (“brain fog” / forgetfulness / poor concentration)	71
Dyspnoea	59
Arthralgia / myalgia	49
Sleep disturbance	34
Depression / anxiety	30
Heart rate dysfunction / palpitations	30
Recurring chest pain	29
Anosmia (loss of smell)	25
Dysgeusia (loss of taste)	25
Low oxygen levels	13

All data were subjected to Shapiro-Wilks normality tests. An unpaired T-test was performed on parametric data with the data expressed as mean ± standard deviation. whereas the Mann–Whitney U test was used on unpaired non-parametric data and the data expressed as median [Q1–Q3] (all two-tailed)

**Fig. 1:**
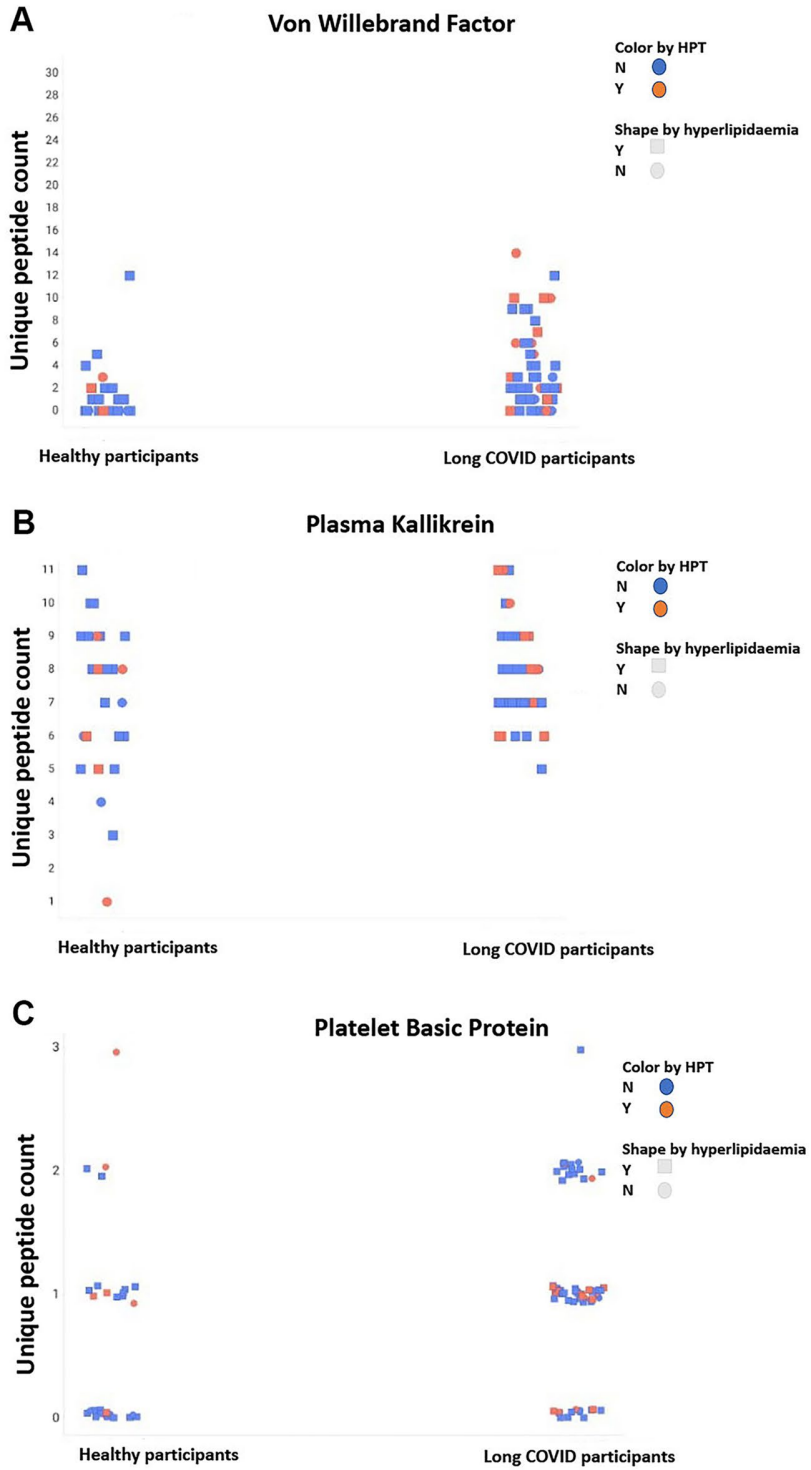
Stripe plots comparing the unique peptide counts of VWF, plasma Kallikrein and PF4 between patients suffering from hypertension and hyperlipidaemia in our long COVID and control population. This demonstrates that there is no correlation between patients suffering from hypertension or hyperlipidaemia and that it doesn’t serve to differentiate between the groups. (Created with BioRender.com.)

We evaluated serum ferritin and IL-6 levels of our high and low responder cohorts and excluded other samples from the analysis. There were no significant differences between long COVID patients and controls in their serum ferritin concentrations. The IL-6 concentrations between high responder long COVID patients and healthy controls were significantly different (see Table 1). We excluded individuals with high Pro-BNP values, as that might point to underlying heart conditions and would influence our results. Some of the most noteworthy molecules we found were VWF, plasma Kallikrein and Platelet Basic Protein or Platelet Factor 4 (PF4.) Fig. 1 depicts stripe plots comparing the unique peptide counts of VWF, plasma Kallikrein and PF4 between patients suffering from hypertension and hyperlipidaemia in our long COVID and control population. This demonstrates that there is no correlation between patients suffering from hypertension or hyperlipidaemia and that it does not serve to differentiate between the groups.

We present our proteomics results as fold changes in levels of proteins. Changes are shown as fold changes for the most significant proteins for pair-wise comparisons. The most noteworthy (i.e. differentially observed) molecules of the digested microclots from the long COVID patients, versus the equivalent volume of solubilized plasma from healthy controls are listed in Table 2 below. As described in Methods, we divided the sample into high and low responders and here we present the data from only the high responders (N = 66). Low responders were excluded from further data analysis. We also show the fold changes of immunoglobulins discovered in Table 3. The quantity of immunoglobulins, expressed as a percentage, present in 63 of our 66 long COVID high responder samples and not found in the control group, is shown in Table 4. Unfortunately, 3 of the long COVID samples were not included in the data analysis of the immunoglobulins shown in Table 4. We only show the relevant immunoglobulins/molecules that were present in 13% or more of the samples (13% was an arbitrarily chosen minimum cut-off).

**Table 2:** Molecules of interest trapped inside the digested microclots of high-responder long COVID patients (N = 66) vs all controls (N = 29)

Uniprot ID	Molecule	Uniprot description / function if available (as specified on Uniprot website or references in the column)	Fold change	p-Value
*Molecules of interest involved in the coagulation system found to be INCREASED in the long COVID sample vs healthy sample*				
P04275	VWF_HUMAN / von Willebrand factor	Important in the maintenance of haemostasis. *It promotes adhesion of platelets to the sites of vascular injury by forming a molecular bridge between sub-endothelial collagen matrix and platelet-surface receptor complex GPIb-IX-V.* Also acts as a chaperone for coagulation factor VIII delivering it to the site of injury stabilizing its heterodimeric structure and protecting it from premature clearance from plasma	2.6	9.4 × 10^− 11^
P02775	CXCL7_HUMAN / Platelet basic protein (LA-PFA4)	LA-PF4 stimulates DNA synthesis, mitosis, glycolysis, intracellular cAMP accumulation, prostaglandin E2 secretion, and synthesis of hyaluronic acid and sulfated glycosaminoglycan. *It also stimulates the formation and secretion of plasminogen activator by human synovial cells*	3.5	3.5 × 10^− 05^
P08697	A2AP_HUMAN / α(2)-antiplasmin	Serine protease inhibitor. *The major targets of this inhibitor are plasmin and trypsin*, but it also inactivates matriptase-3/TMPRSS7 and chymotrypsin	1.3	0.0098 (Marginally increased)
*Molecules of interest involved in the coagulation system found to be REDUCED in the long COVID sample vs healthy sample*				
P03952	KLKB1_HUMAN / Plasma kallikrein	The enzyme cleaves Lys-Arg and Arg-Ser bonds. *It activates in a reciprocal reaction, factor XII after its binding to a negatively charged surface.* It also releases bradykinin from HMW kininogen and may also play a role in the renin-angiotensin system by converting prorenin into renin	4.4	4.2 × 10^− 07^
*Molecules of interest involved in cellular function found to be INCREASED in the long COVID sample vs healthy sample*				
Q08380	LG3BP_HUMAN / Galectin-3-binding protein	Promotes integrin-mediated cell adhesion. *May stimulate host defence against viruses and tumour cells*	2.3	3.9 × 10^− 07^
P07996	TSP1_HUMAN /Thrombospondin-1	*Adhesive glycoprotein that mediates cell-to-cell and cell-to-matrix interactions. Binds heparin*. May play a role in dentinogenesis and/or maintenance of dentin and dental pulp Ligand for CD36 mediating antiangiogenic properties. Plays a role in ER stress response via its interaction with the activating transcription factor 6 alpha (ATF6) which produces adaptive ER stress response factors (by similarity.)	2.4	5.1 × 10^− 05^
P19652	A1AG2_HUMAN / Alpha-1-acid glycoprotein 2	Functions as transport protein in the blood stream. Binds various hydrophobic ligands in the interior of its beta-barrel domain. Also binds synthetic drugs and influences their distribution and availability. *Appears to function in modulating the activity of the immune system during the acute-phase reaction*	2.8	2.5 × 10^− 25^
P19827	ITIH1_HUMAN / Inter-alpha-trypsin inhibitor heavy chain H1	May act as a carrier of hyaluronan in serum or as a binding protein between hyaluronan and other matrix protein, including those on cell surfaces in tissues to regulate the localization. synthesis and degradation of hyaluronan which are essential to cells undergoing biological processes. *Contains a potential peptide which could stimulate a broad spectrum of phagocytotic cells*	2.1	1.4 × 10^− 17^
P19823	ITIH2_HUMAN / Inter-alpha-trypsin inhibitor heavy chain H2	May act as a carrier of hyaluronan in serum or as a binding protein between hyaluronan and other matrix protein, including those on cell surfaces in tissues to regulate the localization, synthesis and degradation of hyaluronan which are essential to cells undergoing biological processes	1.5	8.7 × 10^− 09^
*Molecules of interest involved in cellular function found to be REDUCED in the long COVID vs healthy sample*				
Q8TDL5	LPLC1_HUMAN / Long palate, lung and nasal epithelium carcinoma-associated protein 1	*May play a role in innate immunity in mouth, nose and lungs. Binds bacterial lipopolysaccharide (LPS) and modulates the cellular responses to LPS*	4.7	0.0009
P02788	TRFL_HUMAN / Lactotransferrin	*Transferrins are iron binding transport proteins which can bind two Fe*^*3*+^ *ions* in association with the binding of an anion	2.3	0.006
Q15848	ADIPO_HUMAN / Adiponectin	*Important adipokine involved in the control of fat metabolism and insulin sensitivity, with direct anti-diabetic, anti-atherogenic and anti-inflammatory activities*. Stimulates AMPK phosphorylation and activation in the liver and the skeletal muscle, enhancing glucose utilization and fatty-acid combustion. Antagonizes TNF-alpha by negatively regulating its expression in various tissues such as liver and macrophages and also by counteracting its effects, Inhibits endothelial NF-kappa-B signalling through a cAMP-dependent pathway. May play a role in cell growth, angiogenesis and tissue remodelling by binding and sequestering various growth factors with distinct binding affinities, depending on the type of complex, LMW, MMW or HMW	4.9	5.1 × 10^− 05^
P02763	A1AG1_HUMAN / Alpha-1-acid glycoprotein 1	Functions as transport protein in the blood stream. Binds various ligands in the interior of its beta-barrel domain. Also binds synthetic drugs and influences their distribution and availability in the body. *Appears to function in modulating the activity of the immune system during the acute-phase reaction*	5.2	5.0 × 10^− 28^
*Molecules of interest involved in lipid metabolism found to be INCREASED in the long COVID sample vs healthy sample*				
P02655	APOC2_HUMAN / Apolipoprotein C-II	Component of chylomicrons, (VLDL) very-low-density lipoproteins, low-density lipoproteins (LDL) and high-density lipoproteins (HDL) in plasma. Plays an important role in lipoprotein metabolism as an activator of lipoprotein lipase. Both proapolipoprotein C-II and apolipoprotein C-II can activate lipoprotein lipase. Present in normolipidemic individuals, it is mainly distributed in the HDL, whereas in hypertriglyceridemic individuals, predominantly found in the VLDL and LDL	1.7	3.1 × 10^− 06^
*Molecules of interest involved in lipid metabolism found to be REDUCED in the long COVID sample vs healthy sample*				
P02652	APOA2_HUMAN / Apolipoprotein A	May stabilize HDL (high density lipoprotein) structure by its association with lipids and affect the HDL metabolism	5.2	1.4 × 10^− 14^

Fold changes and P values of molecules that were either increased or reduced in the long COVID high responder population compared to controls are shown here (p = 0.012). Descriptions of the function of the molecules are taken from the Uniprot website and added in the description column. Proteomics data was analysed using multiple testing correction and the Benjamini–Hochberg test was performed. The P-value was adjusted to < 0.012, after the Benjamini–Hochberg correction was applied

**Table 3:** Selected molecules of interest trapped inside the digested microclots of high-responder long COVID patients (N = 66) vs all controls (N = 29)

*Immunoglobulin molecules (or fragments thereof) that were found to be INCREASED in the long COVID (high responder) sample vs the healthy sample*			
Uniprot ID	Molecule	Fold change	p-Value
P01876	Immunoglobulin alpha-1 chain C region (IGHA1_HUMAN)	1.5	1.2 × 10^− 05^
A0A0C4DH32	Immunoglobulin heavy chain V-III region GAL (HV320_HUMAN)	1.6	5 × 10^− 05^
P01602	Immunoglobulin kappa chain V-I region HK102 (Fragment) (KV105_HUMAN)	1.6	0.0003
B9A064	Immunoglobulin lambda-like polypeptide 5 (IGLL5_HUMAN)	1.7	2.5 × 10^− 11^
P01717	Immunoglobulin lambda chain V-IV region Hil (LV403_HUMAN)	1.8	1.7 × 10^− 05^
P01763	Immunoglobulin heavy chain V-III region WEA (HV348_HUMAN)	1.9	0.005
P01764	Immunoglobulin heavy chain V-III region TUR (HV323_HUMAN)	2.1	2.4 × 10^− 05^
P04433	Immunoglobulin kappa chain V-III region VG (Fragment) (KV311_HUMAN)	2.4	1.5 × 10^− 13^
P04207	*Immunoglobulin kappa chain V-III region CLL (B chronic lymphocytic leukaemia) *[32]* (KV315_HUMAN)* *CD5- B cell CLL cell population might produce pathologic IgM kappa rheumatoid factor autoantibodies *[33]	2.3	2.9 × 10^− 08^
P01624	*Immunoglobulin kappa chain V-III region POM (plasma membrane)* *(KV315_HUMAN) rheumatoid factor*	2.3	0.01
P04211	Immunoglobulin lambda chain V region 4A (LV743_HUMAN)	2.5	0.003
P06331	Immunoglobulin heavy chain V-II region ARH-77 (HV434_HUMAN)	2.7	0.002
P04430	Immunoglobulin kappa chain V-I region BAN (KV116_HUMAN)	2.8	2.7 × 10^− 06^
P01762	Immunoglobulin heavy chain V-III region TRO (HV311_HUMAN)	2.8	1.0 × 10^− 08^
P01619	Immunoglobulin kappa chain V-III region NG9 (Fragment) (KV320_HUMAN)	2.9	7.3 × 10^− 05^
P01619	Immunoglobulin kappa chain V-III region B6 (KV320_HUMAN)	3.8	4.1 × 10^− 06^
P80748	Immunoglobulin lambda chain V-V region DEL (LV321_HUMAN)	3.8	1.2 × 10^− 08^
P01780	Immunoglobulin heavy chain V-III region JON (HV307_HUMAN)	4.0	0.002
P01772	Immunoglobulin heavy chain V-III region KOL (HV333_HUMAN)	4.2	1.1 × 10^− 05^
P01700	Immunoglobulin lambda chain V-I region HA (LV147_HUMAN)	4.6	2.3 × 10^− 06^
P01767	Immunoglobulin heavy chain V-III region BUT (HV353_HUMAN)	5.0	1.8 × 10^− 06^
P06312	Immunoglobulin kappa chain V-IV region Len (KV401_HUMAN)	5.6	9.9 × 10^− 21^
P01700	Immunoglobulin lambda chain V-I region WAH (LV147_HUMAN)	14.8	2.4 × 10^− 08^
P01593	Immunoglobulin kappa chain V-I region Ni (KVD33_HUMAN)	76.0	4.5 × 10^− 42^

Fold changes and P values of immunoglobulin molecules (or fragments thereof) that were found to be increased in the long COVID high responder population compared to our healthy controls are shown here (p = 0.01). Immunoglobulin molecules are either present or absent and therefore we only listed molecules (or fragments thereof) that were noted to be increased. We excluded immunoglobulin molecules (or fragments thereof) that were reduced compared to our healthy controls. Descriptions of the molecules are taken from the Uniprot website and were added in the description column. After cross referencing each immunoglobulin on THE INTERNATIONAL IMMUNOGENETICS INFORMATION SYSTEM®’s IMGT/LIGM-DB database a datafile containing a description of the immunoglobulin, the nucleotide sequence, other data and literature references can be downloaded. The descriptions listed below were obtained from these data files

**Table 4:** Immunoglobulins that were ONLY found in patients with long COVID and were present in > 13% of 63 of our 66 high responder samples

Citable accession number	Description	%
A0A5C2GW09_HUMAN	IG c1641_light_IGKV1-6_IGKJ4 (Fragment) OS = Homo sapiens OX = 9606 PE = 2 SV = 1	27
A0A5C2G4F7_HUMAN	IGL c3450_light_IGKV1-39_IGKJ1 (Fragment) OS = Homo sapiens OX = 9606 PE = 2 SV = 1	24
A0A7S5C1N4_HUMAN	IGH c2558_heavy_IGHV3-20_IGHD2-2_IGHJ2 (Fragment) OS = Homo sapiens OX = 9606 PE = 2 SV = 1	22
A0A5C2GTV9_HUMAN	IG c945_light_IGKV3-20_IGKJ1 (Fragment) OS = Homo sapiens OX = 9606 PE = 2 SV = 1	21
A0A5C2GZY9_HUMAN	IG c1248_light_IGLV1-44_IGLJ2 (Fragment) OS = Homo sapiens OX = 9606 PE = 2 SV = 1	21
A0A7S5EWW2_HUMAN	IGH c2567_heavy_IGHV1-24_IGHD5-18_IGHJ4 (Fragment) OS = Homo sapiens OX = 9606 PE = 2 SV = 1	21
A0A5C2G1R6_HUMAN	IGL c2854_light_IGLV1-40_IGLJ2 (Fragment) OS = Homo sapiens OX = 9606 PE = 2 SV = 1	19
A0A7S5BZI6_HUMAN	IGH c1057_heavy_IGHV3-11_IGHD4-23_IGHJ5 (Fragment) OS = Homo sapiens OX = 9606 PE = 2 SV = 1	19
A0A5C2FVE3_HUMAN	IGL c574_light_IGKV3-15_IGKJ5 (Fragment) OS = Homo sapiens OX = 9606 PE = 2 SV = 1	17
A0A5C2FZT4_HUMAN	IGL c1790_light_IGKV3-20_IGKJ3 (Fragment) OS = Homo sapiens OX = 9606 PE = 2 SV = 1	17
A0A5C2G099_HUMAN	IGL c977_light_IGKV3-20_IGKJ3 (Fragment) OS = Homo sapiens OX = 9606 PE = 2 SV = 1	17
A0A5C2G3Y0_HUMAN	IGL c3260_light_IGKV4-1_IGKJ1 (Fragment) OS = Homo sapiens OX = 9606 PE = 2 SV = 1	17
A0A5C2GC79_HUMAN	IGH + IGL c193_light_IGLV3-25_IGLJ3 (Fragment) OS = Homo sapiens OX = 9606 PE = 2 SV = 1	17
A0A5C2GJK9_HUMAN	IG c95_heavy_IGHV3-23_IGHD6-25_IGHJ4 (Fragment) OS = Homo sapiens OX = 9606 PE = 2 SV = 1	17
A0A5C2GVG8_HUMAN	IG c1565_light_IGKV2-28_IGKJ1 (Fragment) OS = Homo sapiens OX = 9606 PE = 2 SV = 1	17
A0A7S5EWD7_HUMAN	IGH c3300_heavy_IGHV3-33_IGHD5-18_IGHJ4 (Fragment) OS = Homo sapiens OX = 9606 PE = 2 SV = 1	17
A0A5C2GFW6_HUMAN	IG c686_heavy_IGHV3-20_IGHD6-25_IGHJ4 (Fragment) OS = Homo sapiens OX = 9606 PE = 2 SV = 1	16
A0A5C2G2C3_HUMAN	IGL c3319_light_IGKV1-8_IGKJ1 (Fragment) OS = Homo sapiens OX = 9606 PE = 2 SV = 1	14
A0A5C2G2K9_HUMAN	IGL c3511_light_IGKV1-9_IGKJ4 (Fragment) OS = Homo sapiens OX = 9606 PE = 2 SV = 1	14
A0A5C2GH36_HUMAN	IG c401_light_IGKV3-20_IGKJ4 (Fragment) OS = Homo sapiens OX = 9606 PE = 2 SV = 1	14
A0A5C2GYP0_HUMAN	IG c860_light_IGKV1D-33_IGKJ2 (Fragment) OS = Homo sapiens OX = 9606 PE = 2 SV = 1	14
A0A7S5C450_HUMAN	IGH c1450_heavy_IGHV3-49_IGHD4-4_IGHJ6 (Fragment) OS = Homo sapiens OX = 9606 PE = 2 SV = 1	14

Descriptions taken from the Uniprot website when a search is done on the accession number

**Fig. 2:**
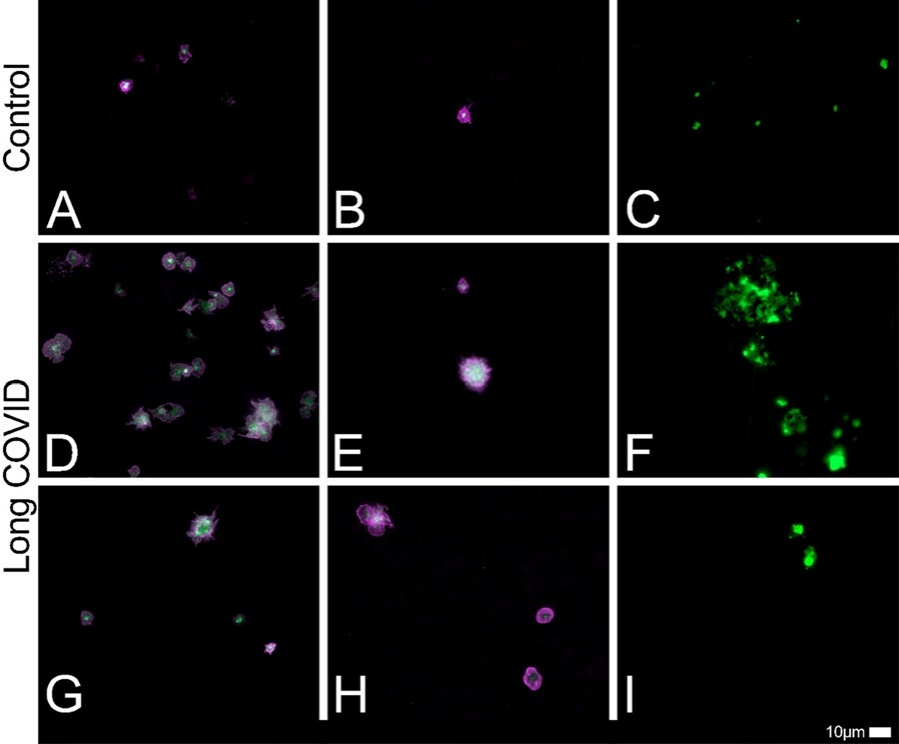
Fluorescence microscopy showing platelets and microclots in individuals with long COVID and control samples. The first 2 columns show platelets in the haematocrit and the last column shows microclots in PPP. PPP were exposed to ThT, a fluorescent amyloid dye (final concentration: 0.005 mM) (Sigma-Aldrich, St. Louis, MO, USA) for 30 min at room temperature and protected from light. The same scoring system can be utilized to evaluate and interpret the fibrinaloid microclot load severity in PPP as published in our previous work [17]. We are aware that this isn’t a perfect system to aid as a quantitative score for qualitative data. Stage 1 would represent a minimal fibrinaloid microclot load as seen in healthy/control PPP, whereas Stage 4 represents a severe fibrinaloid microclot load. The fluorescent micrographs of representative samples of our long COVID cohort and control patients of haematocrit samples are stained with PAC-1 (green fluorescence) and CD62P-PE (purple fluorescence). The white areas represent areas where the two markers overlap, and the images were taken at 63 × magnification. The scoring system as described by Laubscher and Lourens et al. can be used to evaluate platelet activation and clumping in these fluorescence micrographs. In Stage 1 platelets are minimally activated and are seen as small and round with few pseudopodia (representative of healthy/control platelets). Severe platelet activation, or Stage 4 activation, is characterized by large, egg-shaped morphology with aggregations and is indicative of hyperactivated platelets. Similarly, platelet clumping can also be assessed with no clumping seen in healthy/control samples, which is classified as Stage 1 and severe clumping of platelets seen in Stage 4. Therefore, in our long COVID cohort, the fibrinaloid microclot load severity would fit with a severity of stage 2 to 3, and in some instances even a stage 4 (severe microclot load). Mild to moderate platelet activation (stage 2 to 3), as well as clumping are seen in micrographs **D**,** E**,** G** and** H**

Figure 2 shows activated platelets and fibrin amyloid microclots present in representative blood samples from the current cohort. This was in line with the findings of our previous papers [1, 17, 34, 35].

## Discussion

The current proteomics analysis of the content of the fibrin amyloid (plasma protein) microclots we found in our samples, identified increased or decreased levels of inflammatory molecules related to cellular function, coagulation, and lipid metabolism, as well as antibodies trapped inside these microclots. The fluorescent marker used was ThT (demarcated as the green signal in smears of the PPP as seen in Fig. 2). Small areas of protein misfolding will always be present in individuals, for example patients with type II diabetes mellitus, which has been implicated as a protein misfolding disease [36]. However, healthy individuals will also have minor plasma protein misfolding. Long COVID, as a disease, is still so new involves numerous organ systems and affects individuals with numerous comorbidities. In the current paper, we wish to shed more light on the levels of the molecules we found contained within these microclots, albeit increased or decreased and provide researchers and clinicians with a snapshot of the molecules present in patients suffering from long COVID. These molecules may contribute to the symptomatology of patients with long COVID, be involved in the pathophysiology thereof or potentially contribute to autoimmunity in these patients. Hopefully, as researchers piece together the disease pathophysiology, some of the molecules noted in this paper, will give some answers to unravel this very complex condition.

**Fig. 3:**
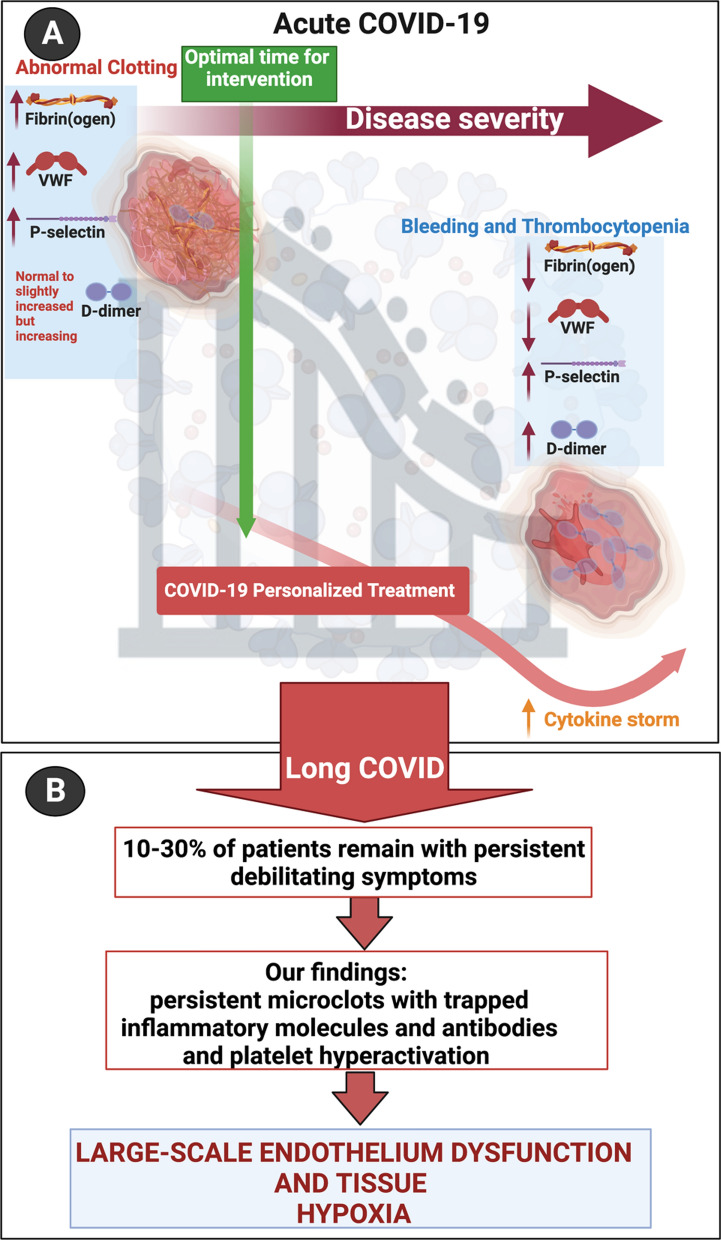
A representation of our understanding of disease progression and pathophysiology from acute COVID-19 to long COVID. **A** Depending on the severity of acute COVID-19 disease, dysregulation with increased levels of the following biomarkers have been found, namely P-selectin [47], fibrinogen, D-dimer and VWF [11]. Abnormal clotting and hypercoagulability are seen early in acute COVID-19 disease with the development of thrombocytopaenia and a risk of bleeding as disease severity progresses. Therefore, the optimal time for intervention is early on in acute COVID-19 disease to address the presence of abnormal clotting. **B** Progression to long COVID disease with patients presenting with debilitating symptoms such as “brainfog,” dyspnoea, chest pain and chronic fatigue and the presence of microclots that may block capillaries with limited passage of red blood cells resulting in reduced O_2_ and CO_2_ exchange. Created with BioRender.com

Many regulatory health bodies still do not recognize long COVID (PASC) as a separate disease entity and refer to it under the broad terminology of “COVID”. There is still a lot to learn about long COVID pathophysiology, but our understanding of the disease is represented in Fig. 3, illustrating the optimal time for intervention and disease progression. From the literature, we do know that many individuals that develop long COVID, are those that might suffer from comorbidities, including hypertension, dyslipidaemia, cardiovascular disease, and previous viral infection(s) [37–40]. Predisposing risk factors or co-morbidities that may also lead to a poor prognosis of acute COVID-19 are well known and include cardiovascular disease, diabetes, arterial hypertension, obesity [12, 13, 41–45], as well as cancer [46].

Individuals with previous reports of autoimmune diseases may also be more prone to develop long COVID. We also know that symptoms that develop during acute COVID infection can become chronic or persistent. We have recently discussed reasons for these persistent symptoms and suggested that it can particularly be ascribed to abnormal blood clotting, a dysfunction in clot lysis, and an entrapment of large numbers of inflammatory molecules inside of fibrinolytic-resistant microclots [35]. These microclots, together with large-scale platelet dysfunction and systemic endothelialitis, may possibly result in generalized cellular hypoxia [17, 35]. Cellular hypoxia, resulting from blockages of the capillaries by microclots might explain most of the persistent (albeit multifarious) symptoms that are noted in long COVID patients. When microclots block capillaries, the passage of red blood cells are limited, resulting in a reduced O_2_ and CO_2_ exchange [35].

We have previously noted that soluble fibrinogen in blood can clot into an anomalous ‘amyloid’ form of fibrin (fibrinaloids), that is comparatively resistant to fibrinolysis [35]. We have also shown that the reason for failed fibrinolysis, might be due to the presence of various molecules entrapped inside the clots, that might prevent clot breakdown [1, 11, 17]. This failed fibrinolysis phenomenon can explain why individuals with long COVID have extensive insoluble fibrinaloid microclots that can persist in circulation, albeit some are trapped in places that are not observed in a standard blood draw. An important consideration is that current pathology tests measure only the soluble inflammatory molecules in blood and standard pathology blood tests such as a full blood count or CRP (C-reactive protein) appear to be normal in these patients. However, if inflammatory molecules are entrapped inside (insoluble) microclots in circulation, the pathology tests will indeed show that the levels of inflammatory molecules are within normal pathology laboratory concentration ranges. From literature we know that IL-6, D-Dimer and fibrinogen levels are elevated in patients with COVID-19, indicating activation of coagulation pathways and a hypercoagulable state or thrombotic risk [48, 49]. The absence of biomarkers in standard pathology tests, result in a significant amount of confusion for patients and clinicians, as patients are extremely sick or even bed-ridden but with no regular identifiable reason for their disease. Biomarkers that are currently available cannot detect the molecules present in the microclots we identified and are therefore unable to confirm their presence or the mechanisms that drive their formation. Further studies are therefore necessary to develop a method or biomarker that would be able to detect and confirm these microclots in patients with long COVID and therefore the appropriate treatment thereof. Our current study, investigating the composition of these microclots, pave the way for future studies to detect their presence.

Our findings that microclots contain and entrap numerous inflammatory molecules, can immediately explain the reasons why traditional pathology tests such as a CRP or full blood count do not find many of the insoluble inflammatory molecules that are and may directly be involved in the disease presentation, pathophysiology and progression [1, 11, 17, 35].

Fibrin-amyloid microclots represent a novel and potentially important target for understanding their inflammatory content and also a target for the treatment of long COVID and related disorders [35]. If we look closely at the entrapped microclot molecules shown in Table 2 that might be directly relevant for cellular function, coagulation, and lipid metabolism we note both increased and decreased levels of molecules when we compare fold changes between healthy individuals and those with long COVID (see Table 2).

### Molecules regulating coagulation

**Fig. 4:**
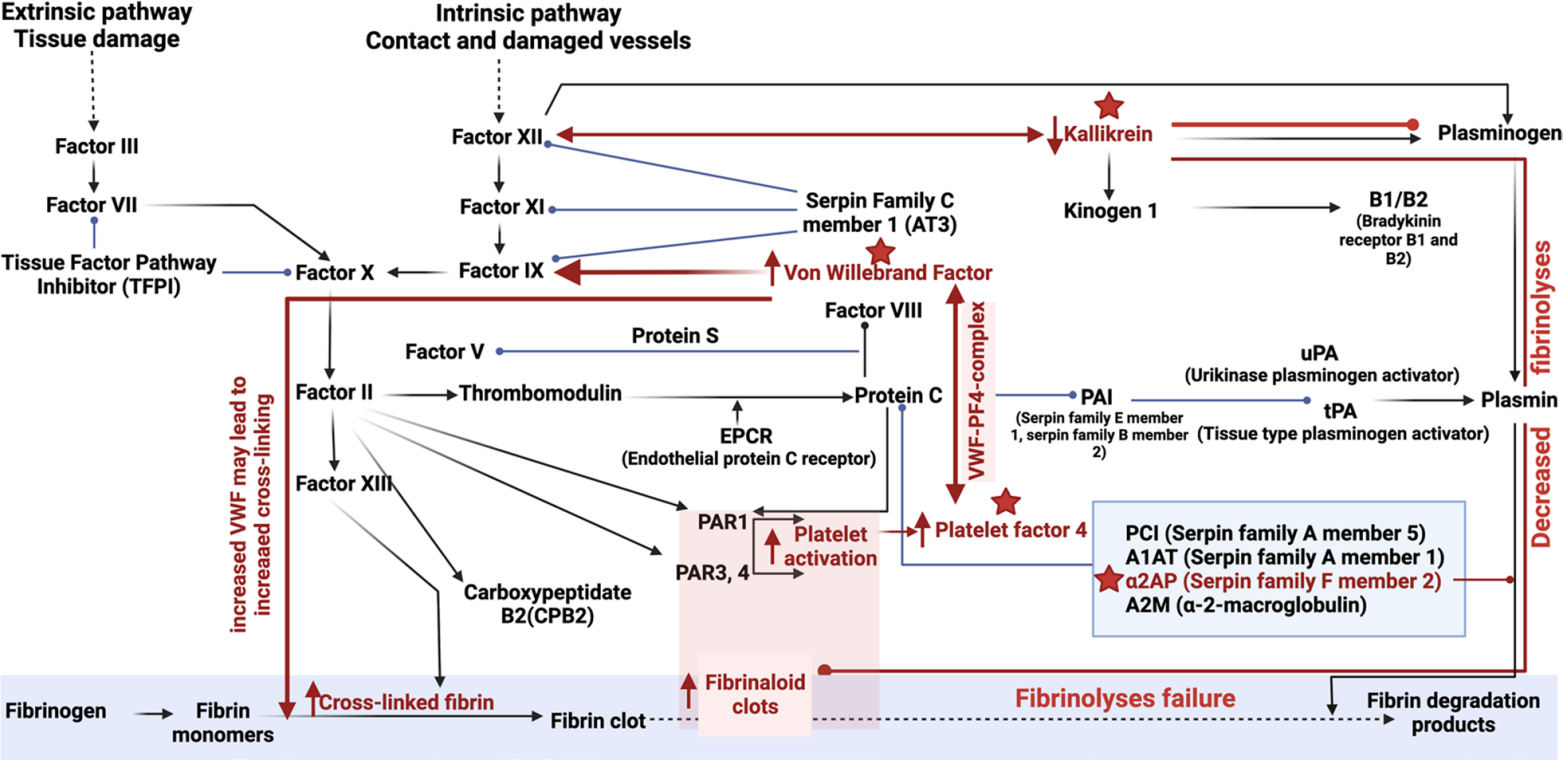
The coagulation pathway to demonstrate the areas of action of the molecules involved in coagulation in our long COVID cohort compared to controls. Activation is illustrated by a line and arrow and inhibition by a line ending in a circular end. The Uniprot ID numbers of our molecules of interest were entered on the David Bioinformatics website and converted to an Entrez_Gene_ID gene list. The generated list was used to search the DAVID Bioinformatics website for molecular pathways where these molecules could potentially play a role. Here we demonstrate the coagulation cascade found on the KEGG pathway database. https://david.ncifcrf.gov/ and https://www.genome.jp/kegg/pathway.html. Diagram created with BioRender.com

We found increased levels of molecules that may modulate coagulation (see Fig. 4 for the areas in the clotting cascade where these molecules will have physiological activity). The most significant ones were VWF and PF4. VWF is a well-known molecule in vascular (endothelial) injury, platelet-platelet adhesion and is generally involved in hypercoagulation, if found to be increased [11, 50] which could contribute to coagulopathies seen in patients with long COVID. PF4 is a small cytokine belonging to the CXC chemokine family that is also known as chemokine (C-X-C motif) ligand 4 (CXCL4). Platelets store PF4, and it is released from the platelet during activation [51]. Heparin can bind to PF4 and promotes PF4 aggregation, resulting in the formation of PF4/heparin complexes, that have antigenic properties [51]. Recently, it was reported that COVID-19 patients might present with reactivity in PF4/heparin antigen tests without the presence of platelet-activating antibodies [52]. PF4 and VWF may also form complexes; recognition of PF4-VWF complexes by heparin-induced thrombocytopenia antibodies contributes to thrombus propagation [53]. Recently, in a smaller cohort of samples, we found that *α-2AP* was increased in long COVID samples [1]. In the current analysis, it was marginally increased. Of significant importance, is that platelets are significantly hyperactivated in long COVID patients. This is noted in both microscopy (as shown in Fig. 2) and the proteomics analysis. Also, it is well-known that individuals with long COVID struggle with anxiety [54, 55]. This was also seen in our long COVID cohort, where 30% of the participants reported anxiety as a symptom (see Table 1). Platelets are well-known for their storage of serotonin (5-HT) [56] and PF4 and serotonin are stored in α- and δ-granules [57]. Circulating immune complexes may also activate platelets via receptor-receptor binding followed by the release of serotonin from platelet granules [57]. Hyperactivated platelets may shed serotonin (and other molecules stored inside platelets), resulting in depletion of serotonin and other molecules stored inside of platelets. The serotonin transporter (SERT or 5HTT) (https://www.ncbi.nlm.nih.gov/books/NBK53687/) is expressed on the plasma membrane of several bodily tissues, for example blood platelets [58, 59], the brain, lungs, bone, gastrointestinal tract as well as the cardiovascular system and transports the neurotransmitter serotonin (5-hydroxytryptamine) across the cell membrane. Prior research suggested that the transport of serotonin (5-HT) via the SERT as well as the storage, metabolism and release mechanisms thereof are similar in platelets and serotonergic neurons. Serotonin depletion may be a significant contributor to the depressed mood or anxiety noted in individuals suffering from long COVID.

**Fig. 5:**
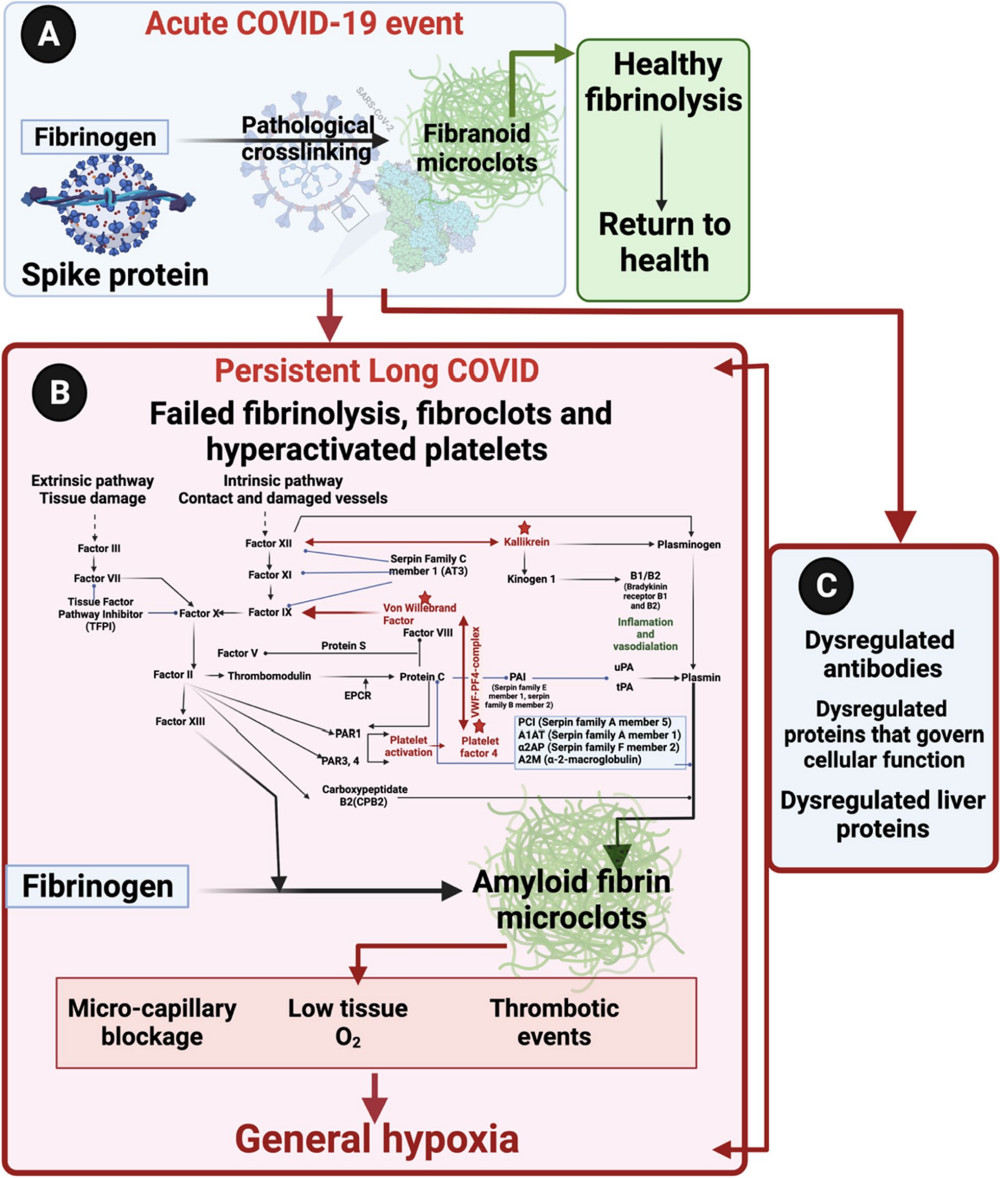
**A** Progression from acute COVID-19 to **B** long COVID and the persistence of microclots and a general hypoxic state, accompanied by increased circulating antibodies, proteins related to cellular function and liver protein dysfunction (**C**)

*Plasma kallikrein (KLKB1)* levels were found to be decreased in the current long COVID cohort, and is known to activate factor XII, and it also belongs to a subgroup of trypsin-like serine proteases, enzymes capable of cleaving peptide bonds in proteins [60]. It is also involved in the bradykinin pathway, that is seen as an inflammatory product of the coagulation system [61]. Both neutrophil and mast-cell activation are functionally linked to the bradykinin pathways [61], and mast cell activation is a known feature of PASC, consistent with the known benefits of antihistamine treatment [62] KLKB1 also digests plasminogen to plasmin and participates in surface-dependent activation of blood coagulation, fibrinolysis, and inflammation. Under physiological conditions, plasma kallikrein serves as a cardioprotective enzyme, but when it is increased in circulation, it is involved in cardiovascular disease [63]. A decrease in the level of this molecule may prevent adequate plasminogen activation and may directly impact downstream on clot breakdown and therefore fibrinolysis (see Fig. 5.)

### Molecules regulating cellular function

Molecules that modulate cellular function that we found to be increased, included *galectin-3-binding protein, Thrombospondin-1, Alpha-1-acid glycoprotein-2 as well as Inter-alpha-trypsin inhibitor heavy chain H1 and 2 (ITIH1 and ITIH2)*. *Galectin-3* expression is known to be induced in viral infections and by a multitude of molecules that either mimic or are characteristic of ongoing inflammation and microbial infection, such as IFN-α, IFN-β, IFN-γ, TNF-α, poly(I:C), dsRNA, and dsDNA [64]. In addition, it is commonly overexpressed in most types of cancers [65] and is an important player in cancer development, progression, and metastasis via its interactions with galactoside-terminated glycans [66]. Furthermore, it interacts with the cell-surface glycoprotein CD146 (MCAM, MUC18) and induces secretion of metastasis-promoting cytokines from vascular endothelial cells [66]. Recently, it was also identified as a novel predictor of venous thromboembolism in systemic lupus erythematosus [67].

*Thrombospondin-1* was also found to be increased in our cohort of long COVID participants. It is an adhesive glycoprotein that is released from platelet alpha-granules in response to thrombin stimulation and is also a transient component of the extracellular matrix in developing and repairing tissues [68]. Recently it was suggested that it might regulate haemostasis in vivo through modulation of platelet cAMP signalling at sites of vascular injury [69], and that it could mark a prothrombotic state and an underlying inflammatory process [70]. It is also increased in the tumour microenvironment [64] and might be involved in antiphospholipid syndrome [70], a condition well known to be associated with thrombosis.

*Alpha-1-acid glycoprotein-2*, which we also found to be elevated, is known to modulate the immune system during the acute-phase reaction. *ITIH1 and ITIH2* were found to be elevated in our long COVID population. These molecules belong to the inter-α-trypsin inhibitors. Previously, it was reported that ITIH1 and ITIH2 were more abundant in a COVID-19 survivor group [71]. ITIH1 has previously been found to be elevated in patients with osteoarthritis [72]. ITIH1 is synthesized by chondrocytes and binds to hyaluronic acid and also to the extracellular matrix [73]. ITIH2 has been associated with demyelinating diseases [74].

We found decreased levels of a few molecules, namely *Long palate, lung and nasal epithelium carcinoma-associated protein 1 (LPLC1_HUMAN,) Lactotransferrin, Adiponectin* and *Alpha-1-acid glycoprotein 1 (A1AG1_HUMAN)* that might point to a type of immunosuppression. Our research group also suggested that Lactoferrin may be beneficial for preventing adverse effects of acute COVID-19, mainly due to its antiviral properties [75]. Previously, it was found that some critically ill COVID-19 patients could not be discharged from the ICU even though they exhibited negative viral tests [76]. Zhou and co-workers in 2021 suggested that in such patients, adaptive immunosuppression might be present, and ascribed it to a dysregulated host response to severe infection, similar to the immunosuppression that exists in patients with sepsis [76]. In long COVID, there has also been reports of immune suppression [54].

Of importance may be *LPLC1,* which may play an important role in innate immunity, as its function is related to binding of LPS, as a LPS-binding protein (https://www.uniprot.org/uniprot/C3TTP7). If LPS is not bound effectively, it may have significant implications for the response to LPS in circulation. This could possibly indicate an altered immune response to infectious pathogens. Related to LPS binding, is *Lactotransferrin* also known as lactoferrin (LF,) that was also decreased in our long COVID cohort. LF has important immunological properties and is both antibacterial and antiviral. In particular, there is evidence that it can bind to at least some of the receptors used by coronaviruses and thereby block their entry [75]. LF makes use of Heparan Sulfate Proteoglycans (HSPGs) on the surface of epithelial cells of the stomach to facilitate entry into the bloodstream via endocytosis [77, 78]. Coronaviruses bind to host cells by first attaching to HSPGs utilizing them as preliminary docking sites to assist with viral entry and LF is able to interfere with some of the receptors used by coronaviruses. LF can inhibit the entry of viral particles into host cells by blocking cellular receptors or via direct attachment to viral particles. In COVID-19 infection, LF might be able to competitively bind receptors as well as HSPGs and therefore prevent viral entry and the subsequent cytokine storm [75] but this still requires further investigation. Our finding of decreased levels of lactoferrin possibly indicates suppression of the host immune response in patients with long COVID.

*Adiponectin* has anti-inflammatory and anti-apoptotic properties that has shown to protect the vasculature, heart, lung, and colon [79], and was also reduced in our long COVID population. Pro-inflammatory and anti-inflammatory cytokines or adipokines namely adiponectin, leptin and resistin may be released from adipose tissue [80]. Adiponectin exists in two isoforms namely HMW (high molecular weight) and LMW (low molecular weight) adiponectin and is an important regulator of cytokine immune responses. These cytokine responses are isoform specific. IL-6 secretion by human monocytes and human monocytic leukemia cell line cells are stimulated by HMW adiponectin and does not inhibit LPS-induced IL-6 secretion. On the other hand, LMW adiponectin promotes IL-10 secretion and inhibits LPS-mediated IL-6 release. For example, in chronic hepatitis C infection these cytokines play an important role in the inflammatory and immune response of affected individuals. This could explain the different outcomes seen in hepatitis C infection. Some of the metabolic and inflammatory effects of adiponectin are regulated through the mitogen-activated protein kinase (MAPK) signalling pathway [80]. The MAPK pathway forms a crucial part in the regulation of IFN-α and INF-γ signalling in viral infections such as hepatitis C. The reduced level of adiponectin in our long COVID cohort also potentially points to an altered immune response in infected patients.

*Alpha-1-acid glycoprotein 1* is an acute-phase protein [81] and functions as a transport protein in the blood stream, and it was found to be reduced in our long COVID participants. It is known to regulate inflammation, including binding of pathogens and modulating white blood cells’ activity throughout the entire leukocyte attacking sequence [82]. This molecule is also known to modulate the immune system during the acute-phase reaction and decreased levels are related to the mortality of septic patients [83]. The decreased levels seen in the current cohort, might possibly indicate a dysregulated or abnormal immune or inflammatory response. It may therefore also be a predictor of morbidity and mortality in patients with COVID-19. If we look at the physiological processes that are regulated by the aforementioned molecules, the increased or reduced levels of these molecules will have an effect on cellular function and the potentially dysregulated immune response in these patients. They also have immune-related or cellular remodelling properties and may impact on vascular function.

### Molecules regulating lipid metabolism and regulation

We also found interesting apolipoproteins, known to play a role in lipid metabolism and regulation. *Apolipoprotein C-II* (a component of very low density lipoproteins and chylomicrons) was found to be increased while *Apolipoprotein A-II* (the second most abundant protein of the high density lipoprotein particles) was reduced in our cohort. Previous studies support the crucial role that lipids and lipid metabolism play in SARS CoV-2 infection [84–86]. Shen et al. performed proteomic and metabolomic profiling on the serum of 46 COVID-19 patients compared to 53 controls [84]. They found the downregulation of over 100 lipids, including sphingolipids, glycerophospholipids and fatty acids which correlated with disease severity in patients with COVID-19. Lipids, including sphingolipids and glycerophospholipids form an essential part of biomembranes. Specialized membrane domains exist in the plasma membrane and sphingolipids are an example of this. Sphingolipids are also involved in the regulation of signal transduction, immune activation pathways and inflammatory responses [87]. The downregulation of lipids in the sera of patients suffering from COVID-19 points to liver damage and changes in bilirubin and bile acids supports this. Song et al. studied the lipidome and metabolome of 50 patients with different severities (mild, moderate, and severe) of SARS CoV2 infection compared to 26 healthy controls by utilizing targeted and untargeted mass spectrometry of plasma samples that were collected [85]. They noted reductions of major classes of plasma glycerophospholipids, phosphatidic acids (PAs), phosphatidylinositols (PIs), and phosphatidylcholines (PCs) and increased concentrations of lysophospholipids namely lysophosphatidic acids (LPAs,) lysophosphatidylinositols (LPIs,) and lysophosphatidylcholines (LPCs.) Another finding was that the plasma lipidome of patients with COVID19 had similarities to monosialodihexosyl ganglioside (GM3)-enriched exosomes where levels of sphingomyelins (SMs) and GM3s are elevated, and diacylglycerols (DAGs) decreased. Increasing levels of GM3s were seen with progressive disease severity indicating that GM3-rich exosomes also play a role in the pathophysiology of COVID19.

From the literature it is evident that fluctuations in DGs (diglycerides,) FFAs (free fatty acids), and TGs (triglycerides) occur in pathological conditions. For example, increased lipolysis of adipose tissue is seen in Ebola virus disease where TGs are converted to FFAs and DGs, with increased recycling of fatty acids back into TGs [88, 89]. Bruzzone et al. found elevated levels of serum TG, TG-VLDL (VLDL, very low-density lipoproteins), TG-IDL (IDL, intermediate density lipoproteins), TG-LDL (LDL, low-density lipoproteins) and TG-HDL (HDL, high density lipoproteins) in patients with COVID-19 in contrast to a decreased total cholesterol (TC) concentration. They propose that serum accumulation of TG and TG-VLDL may indicate decreased oxidation of acetyl-CoA in the mitochondria of the liver. Ketone bodies are produced by the liver from fatty acid oxidation-derived acetyl-CoA [90]. This pathogenic redistribution of the lipoprotein particle size and composition increases the cardiovascular risk for patients with COVID-19 infection.

Literature supports the role that apolipoproteins and steroid hormones have to play in SARS CoV-2 infection. Shen and co-workers in 2020 [84] found decreased levels of apolipoproteins APOA1, APOA2, APOH, APOL1, APOD, and APOM in the serum of patients following SARS CoV2 infection. It has also previously been reported that APOA1 levels decreased when patients progressed from mild to severe disease [91]. These apolipoproteins are involved in the functioning of macrophages [92]. Arachidonic acid (AA) is an essential fatty acid. Prostaglandins, leukotrienes and thromboxanes are pro-inflammatory metabolites of AA and eicosapentaenoic acid (EPA). Lipoxins, resolvins, protectins and maresins are derivates of AA, EPA and docosahexaenoic acid (DHA) and are able to suppress the inflammatory response, enhance phagocytosis of macrophages as well as other immune cells and assist with microbial clearance [92, 93]. Therefore, lipids such as glycerophospholipids and fatty acids play a pivotal role in inflammation and the host immune response against viral infections.

In summary, lipid metabolism plays an integral role in COVID-19 disease, resulting in an imbalance between immune-regulating lipids, pro-inflammatory lipids and lipid mediators during the host immune response. It is evident that our findings of increased levels of *Apolipoprotein C-II* and reduced levels of *Apolipoprotein A-II* also supports this dysregulation of lipid metabolism in patients suffering from COVID-19 (see Fig. 5).

### Immunoglobulins of interest

Table 3 shows selected immunoglobulins involved in the immune response and that might act as antibodies or autoantibodies. As can be noted in Table 3, there are numerous molecules with no data available on their function. However, we do list these molecules here, as it may be of value for immunologists to note the presence of these potentially important antibodies. Of specific interest was the presence of *Immunoglobulin kappa chain V-III region POM* (plasma membrane), that is known as rheumatoid factor (RF) and that may act as an autoantibody [33]. Another RF factor that was found to be elevated was *Immunoglobulin kappa chain V-III region CLL.*

Of significance also, was that there were numerous antibodies only present in 63 of our long COVID high responder samples and we present those in Table 4 that were present in 13% or more (arbitrary chosen cut-off) of the cohort. *IG c1641_light_IGKV1-6_IGKJ4 (Fragment), IGL c3450_light_IGKV1-39_IGKJ1 (Fragment) and IGH c2558_heavy_IGHV3-20_IGHD2-2_IGHJ2 (Fragment)* were found to be present in 27%, 24% and 22% of the 63 samples respectively. These antibodies were cross referenced on THE INTERNATIONAL IMMUNOGENETICS INFORMATION SYSTEM®’s IMGT/LIGM-DB database, but no data are available on these antibodies. These antibodies may have significance in the immune response following acute COVID-19 illness or driving auto-immunity in some long COVID participants.

Another important consideration is that patients with long-term persistent microclots, may also progress to develop auto-immune diseases. We suggest that this phenomenon might directly be due to autoantibodies that develop against antibodies or other molecules that are entrapped inside the fibrinaloid microclots. It appears that the long-term sequelae of many viral infections [94, 95], including long COVID [96, 97] may be attributed to autoimmunity. These microclots contain many different proteins that may present new epitopes if a conformational change has taken place. These epitopes may be recognized as ‘non-self’ eliciting an immune response with the possible development of autoantibodies. Additionally, oxidative stress induces the nitration of proteins leading to the production of autoantibodies. Autoantibodies formed against actin lead to muscle weakness [98]. In ME/CFS (Myalgic Encephalomyelitis/Chronic Fatigue Syndrome), autoimmunity is well described [94]; and might also play an important role in long COVID because some autoantibodies share elements of epitope with the SARS-CoV-2 virus [99]. It has also been suggested that some of the viral sequences are amyloidogenic [100, 101]. Viral persistence may play a key role in patients suffering from long COVID [102], and therefore driving an ongoing immune response or the development of autoimmunity (see Fig. 5).

Pascolini et al. studied 33 consecutive patients with COVID-19 of whom 94% had interstitial pneumonia and compared them to 25 age- and sex-matched controls with fever and/or pneumonia of an etiology other than COVID-19 to evaluate for autoimmune serological markers [103]. 45% of patients with COVID-19 tested positive for at least one autoantibody and they found that 33% tested positive for antinuclear antibodies (ANAs), 24% tested positive for anti-cardiolipin antibodies (immunoglobulin (Ig)G and/or IgM) and 9% tested positive for anti-β2-glycoprotein antibodies (IgG and/or IgM.) Antineutrophil cytoplasmic antibodies (ANCA) were not present in any of their patients. A higher mortality rate was seen amongst patients with autoantibodies compared to those without.

Chang and coworkers searched for autoantibodies in hospitalized patients with COVID-19 and found that approximately 50% of hospitalized patients developed autoantibodies against one or more antigens in the array they tested for with 25% of patients being ANA positive [104]. They also discovered antibodies that are associated with relatively rare connective tissue diseases and have been predicted to be pathogenic. A large number of anti-cytokine antibodies (ACA) were identified as well as antibodies recognizing non-structural SARS-CoV-2 proteins which correlate with autoantibodies. A few of the antibodies they discovered include Anti-C1q, which is a SLE autoantigen, anti-β2GP1 (anti-beta 2 glycoprotein 1) a thrombogenic antibody, anti-BPI (anti-bactericidal permeability inducing protein) and anti-ACE2 antibodies.

Conversely, a study performed by Klein et al. suggest that autoantibodies have a limited role to play in the pathogenesis of long COVID [105]. Their findings point to the involvement of persistent viral antigen, reactivation of latent herpesviruses, and chronic inflammation in long COVID pathophysiology. There have been previous reports of persistent viral antigen in intestinal biopsies of patients recovering from COVID-19 infection and antigen persistence may therefore contribute to the host  immune response in patients with long COVID [106, 107].

## Conclusion

The present proteomics study provides further evidence of the importance of not only looking at the soluble inflammatory molecules present in blood samples of individuals who suffer from long COVID. Insoluble inflammatory molecules and immunoglobulins may be of significant importance in not only causing the persistent long COVID symptoms, but also eventually leading to these individuals developing auto-immune diseases. An important consideration is to resolve these microclots, address platelet hyperactivation and wide-spread vascular inflammation preferably in the acute phase of the disease. In that way auto-immunity and the resulting severe and debilitating symptoms due to these pathologies, might be prevented. In addition, if the microclots are resolved early in the disease, general hypoxia that may lead to irreversible tissue damage, could possibly be prevented. Such pathologies may have significant implications if patients already have co-morbidities such as type II diabetes or cardiovascular disease. There is therefore an urgent need for randomized controlled clinical trials to target the prevention or resolution of these microclots in circulation and reversal of the underlying associated endotheliopathy.

## Data Availability

The datasets generated as well as figure micrographs analyzed during the current study are available on request. The raw data supporting the conclusions of this article will be made available by the authors, without undue reservation.
